# Impact of Seasonal Variation on Antioxidant, Enzyme Inhibitory, and Anti‐Inflammatory Potentials of *Cistus creticus* Leaf Extracts

**DOI:** 10.1002/open.202500362

**Published:** 2025-09-21

**Authors:** Khalil Guelifet, Mokhtar Benmohamed, Khaled Kherraz, Latifa Khattabi, Barbara Sawicka, Ayomide Victor Atoki, Abderrazek Ferhat, Lilya Harchaoui, Mohamed Amine Ferhat, Wafa Zahnit, Afaf Khadra Bendrihem, Mohammed Messaoudi

**Affiliations:** ^1^ Laboratoire de Recherche sur les Produits Bioactifs et Valorisation de la Biomasse Département de Chimie école normale supérieure de Kouba (ENS) B.P. 92, Vieux‐Kouba Alger 16308 Algeria; ^2^ Laboratory of Fundamental Sciences University Amar Télidji of Laghouat Po. Box. 37G, Road of Ghardaïa Laghouat 03000 Algeria; ^3^ Laboratoire d’Ethnobotanique et Substances Naturelles Ecole Normale Supérieure vieux‐Kouba Algiers 16050 Algeria; ^4^ Centre de Recherche en Biotechnologie (CRBt) Ali Mendjli Nouvelle Ville UV 03 BP E73 Constantine 03 BP E73 Constantine Constantine 25000 Algeria; ^5^ Department of Plant Production Technology and Commoditties Science University of Life Sciences in Lublin Akademicka 15 str. Lublin 20‐950 Poland; ^6^ Department of Biochemistry Kampala International University Ishaka Uganda; ^7^ Laboratory of Research in Science and Environment: Bioresources, Geochemistry‐Physics, Legislation and Socio‐Economic Development Faculty of Sciences and Technology University of Tamanghasset B.P. 10034, Sersouf Tamanrasset Algeria; ^8^ Faculty of Biological Sciences University of Science and Technology Houari Boumediene Algiers 16111 Algeria; ^9^ Department of Chemistry Faculty of Sciences University of Ferhat ABBAS Setif 1 El Bez 19000 Algeria; ^10^ Biotechnology, Water, Environment and Health Laboratory Faculty of Natural and Life Sciences University of Abbes Laghrour Khenchela 40000 Algeria

**Keywords:** anti‐inflammatory, antimicrobial activity, antioxidant activities, *Cistus creticus*, enzyme inhibition, phytochemicals, seasonal variations

## Abstract

This study investigates the influence of seasonal variation onthe phytochemicalcomposition and biological activities of *Cistus creticus* leaf extracts collected during spring, summer, autumn, and winter. Extracts are analyzed for phenolic and flavonoid contents and evaluated for antioxidant, enzyme inhibitory, anti‐inflammatory, analgesic, antimicrobial, and photoprotective properties. Pronounced seasonal differences are observed. Spring and summer extracts, enriched in bioactive compounds, exhibit the strongest pharmacological potential, including notable antioxidant effects, potent enzyme inhibition, and high photoprotective capacity. The spring extract further demonstrates significant in vivo anti‐inflammatory and analgesic effects, while the winter extract displays superior in vitro anti‐inflammatory activity. These findings highlight the critical role of harvest season in modulating both phytochemical composition and bioefficacy. The superior performance of spring and summer extracts underscores the potential of *C. creticus* as a valuable natural source of antioxidants, enzyme inhibitors, and photoprotective agents. Overall, this work supports the strategic use of seasonal optimization to enhance the therapeutic and cosmeceutical applications of *C. creticus*.

## Graphical abstract

The phytochemical profile and biological activities of *Cistus creticus* leaf ethanolic extracts show strong seasonal variation. Spring extracts contain the highest phenolic levels with potent antioxidant and anti‐inflammatory effects, while summer extracts are richest in flavonoids and exhibit the greatest sun protection factor value and enzyme inhibitory activities. Winter extracts demonstrate superior anti‐inflammatory efficacy in vitro through bovine serum albumin denaturation testing. Overall, spring and summer are optimal harvest seasons for obtaining bioactive‐rich extracts with promising dermocosmetic andphytopharmaceutical potential.

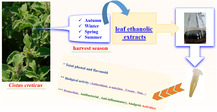

## 1 Introduction

Phenolic compounds are among the most prominent secondary metabolites in plants, known for their broad spectrum of biological activities and increasing importance in medical and pharmaceutical research.^[^ ^1^ ^]^ These molecules play a pivotal role in mitigating chronic noncommunicable diseases such as cancer, diabetes, cardiovascular disorders, and neurodegenerative conditions, primarily through their capacity to modulate oxidative stress, neutralize reactive oxygen species, and influence key signaling pathways involved in inflammation and apoptosis.^[^ ^2^ ^]^ In plants, phenolics are synthesized as part of adaptive defense mechanisms against abiotic and biotic stressors including ultraviolet radiation, pathogen invasion, and herbivory making medicinal plants rich reservoirs of such bioactive constituents.^[^ ^3^ ^]^ Within this context, *Cistus creticus* L., commonly known as ‘rock rose’ or ‘Cretan cistus,’ is an aromatic and medicinal shrub belonging to the family Cistaceae. This genus includes more than fifty evergreen species widely distributed across the Mediterranean Basin, particularly in southern Europe, North Africa, and western Asia.^[^ ^4^ ^]^ In Algeria, *C. creticus* is found naturally in mountainous and rocky habitats under coastal and semi‐arid Mediterranean climates.^[^ ^5^ ^]^ The plant is morphologically characterized by its shrubby form, pink to violet flowers, and glandular trichomes on the leaves that secrete fragrant, biologically active compounds.^[^ ^6^ ^]^

Traditionally, *C. creticus* has been used in Mediterranean folk medicine for managing dermatological conditions (e.g., eczema, abscesses), gastrointestinal disorders, and hair loss.^[^ ^7^ ^]^ In Morocco, its leaf decoctions are employed as a remedy for diabetes.^[^ ^8^ ^]^ The plant's resin, known as labdanum, has been historically valued not only for its medicinal uses but also in perfumery and incense. The therapeutic potential of *C. creticus* is largely attributed to its abundance of labdane‐type diterpenes, flavonoids, and phenolic acids, which have demonstrated antioxidant, anti‐inflammatory, antimicrobial, anticancer, and skin‐protective activities.^[^ ^9^ ^]^

However, the biosynthesis and accumulation of these secondary metabolites are not static; they are influenced by several environmental and biological variables—including geographic origin, developmental stage, and, critically, seasonal variation. Changes in temperature, photoperiod, solar irradiance, and soil moisture across seasons can markedly alter a plant's metabolic profile, affecting both the quantity and bioactivity of its constituents.^[^ ^10^ ^]^ Despite its traditional and pharmacological relevance, *C. creticus* remains underinvestigated in terms of seasonal dynamics and their influence on therapeutic efficacy.

The present study aims to comprehensively evaluate the influence of seasonal variations on the phytochemical profile (phenolic and flavonoid content) and spectrum of biological activities (including antioxidant, anti‐inflammatory, antimicrobial, and enzymatic activities) of ethanolic extracts of *Cistus creticus*. By considering the seasonal dimension, this work aims to deepen the understanding of how environmental factors modulate the bioactivity of the plant, which will consequently contribute to the development of optimized harvesting, extraction, and formulation strategies for pharmaceutical and nutraceutical applications, thus increasing the value of this underexplored medicinal resource.

In the case of the study of the content of phenolic compounds (total phenolic content (TPC)) and flavonoids (total flavonoid content (TFC)) in *Cistus creticus* extracts in different seasons, the following null hypothesis is proposed: 1) there are no statistically significant differences in the TPC and TFC in ethanolic extracts of Cistus creticus collected in different seasons. 2) Ethanolic extracts of Cistus creticus collected in spring are characterized by the highest content of phenolic compounds (TPC), while extracts collected in summer show the highest content of flavonoids (TFC). The content of both these groups of compounds is statistically significantly lower in winter extracts.

## 2 Results and Discussion

### 2.1 Total Phenolic and Flavonoid Content Estimation

Phenolic and flavonoid compounds are among the most important plant constituents due to their ability to scavenge free radicals, a property attributed to their hydroxyl groups. The total phenolic and flavonoid contents of the plant extracts were determined using equations derived from calibration curves of gallic acid and quercetin, respectively. The results are presented in **Table** **1**.

**Table 1:** Total phenolic and flavonoid contents of *Cistus creticus* ethanolic leaf extracts across four seasons.

Specificationa)	TPC [µgGAE mg^−1^]	TFC [µgQE mg^−1^]	SE
C Spring	662.92^a^ ± 0.76	242.00^b^ ± 4.00	2.30
C Winter	569.46^c^ ± 2.40	168.77^c^ ± 0.94	0.54
C Autumn	607.92^b^ ± 5.66	250.22^b^ ± 1.17	0.67
C Summer	596.89^b^± 2.83	271.33^a^ ± 1.88	1.88
LSDp_0.05_	27.30	13.05	–
Mean	609.30	233.08	–

a) Values are expressed as mean ± SD (*n* = 3). Different superscript letters within the same column indicate significant differences among seasons according to ANOVA followed by LSD test at *p* < 0.05. SE: Standard Error.

#### 2.1.1 TPC Analysis

The analysis revealed important seasonal fluctuations in both phenolic and flavonoid accumulation.

Spring (C Spring): The spring extract has the highest phenolic content (662.92 ± 0.76 µgGAE mg^−1^). The “a” designation suggests that this value is statistically higher than the phenolic content in the other seasons (winter, autumn, summer). This may indicate that the plant is intensively synthesizing phenolic compounds in the spring period, probably in response to increased growth or increased exposure to environmental factors after winter.

Autumn (C Autumn) and Summer (C Summer): The extracts obtained in autumn (607.92 ± 5.66 µgGAE mg^−1^) and summer (596.89 ± 2.83 µgGAE mg^−1^) have similar and intermediate TPC values, marked with the letter “b”. This means that there are no statistically significant differences between them, but both are lower than the spring content and higher than the winter content. This suggests that phenolic levels remain at a stable, high level during these seasons.

Winter (C Winter): The winter extract showed the lowest phenolic content (569.46 ± 2.40 µgGAE mg^−1^), marked with a “c”, indicating a statistically significant difference compared to the other seasons. This decrease is likely related to the lower metabolic activity of the plant during the winter dormancy period.

To summarize TPC: Spring is the peak of phenolic compound (TPC) production, which may be crucial for the use of *Cistus creticus* in applications requiring high concentrations of these compounds.

This seasonal pattern can be attributed to several physiological and environmental factors. During spring, increased photosynthetic activity, optimal temperature conditions, and longer photoperiods stimulate the phenylpropanoid pathway, leading to enhanced phenolic biosynthesis. Additionally, spring represents a period of active growth and metabolic activity, during which plants invest heavily in secondary metabolite production for defense against emerging pathogens and herbivores.^[^ ^11^ ^]^

#### 2.1.2 TFC Analysis

Summer (C Summer): The summer extract showed the highest flavonoid content (271.33 ± 1.88 µgQE mg^−1^), marked with a “a”. This is statistically the highest value among all seasons. This indicates that summer is the optimal period for flavonoid accumulation, perhaps due to the intense solar radiation, as flavonoids play a protective role against UV.

Spring extracts (242.00 ± 4.00 µgQE mg^−1^) and autumn extracts (250.22 ± 1.17 µgQE mg^−1^) had similar and intermediate TFC values, marked with the letter "b". There are no statistically significant differences between them, but both of these values are lower than the summer content and higher than the winter one.

Winter (C Winter): Similar to TPC, the winter extract is characterized by the lowest flavonoid content (168.77 ± 0.94 µgQE mg^−1^), marked with the letter “c”, which indicates a statistically significant decrease.

To sum up, TFC: Summer is the most favorable period for obtaining flavonoids from *Cistus creticus.*

Overall Conclusion: Seasonal Dependence: The results obtained confirm that the harvest time of *Cistus creticus* has a key influence on the content of phenolic compounds and flavonoids in ethanol extracts. The accumulation of these bioactive metabolites was dynamic and related to the life cycle of the plant and environmental conditions. Optimal Harvest Times: To obtain an extract rich in total phenolics, the best season seems to be spring.

Contradictory results exist, likely attributable to both the absence of standardization and the significant variability in phytoconstituents. The variables are influenced by various factors, including the preparation methods of plant extracts, seasonal harvesting, drought stress, and soil conditions.^[^ ^12^ ^]^

The peak in summer flavonoid content likely reflects the plant's adaptive response to increased UV radiation and heat stress. Flavonoids serve as natural sunscreens, protecting plant tissues from photodamage while maintaining photosynthetic efficiency.^[^ ^13^ ^]^ The elevated temperatures and intense solar radiation characteristic of Mediterranean summers trigger enhanced flavonoid biosynthesis as a protective mechanism.^[^ ^13^ ^]^

In addition to these drivers, seasonal variation in flavonoid levels may also be modulated by other abiotic factors, including water availability, soil conditions, and plant phenological stage. This multifactorial perspective, supported by reports linking diverse environmental stresses to secondary metabolite accumulation,^[^ ^14^ ^]^ suggests that the observed summer increase results from the combined action of several ecological pressures rather than a single dominant factor. Future studies that integrate phytochemical profiling with precise environmental measurements will help disentangle the relative contribution of each variable.

When compared with previous studies, notable differences emerge, reflecting the influence of several variables. Kilic et al.^[^ ^15^ ^]^ examined the total phenolic content of *Cistus creticus* extracts collected in May, reporting 130.32 µg GAE mg^−1^ and total flavonoid content of 83.94 µg QE mg^−1^—both values markedly lower than those recorded in our study, possibly due to differences in harvest time or sample characteristics.

In another study conducted by Waed et al.^[^ ^16^ ^]^ on *Cistus* species in Syria, considerable amounts of phenolics and flavonoids were detected in methanolic extracts, with values of 69.34 mg GAE g^−1^ and 11.56 mg rutin g^−1^, respectively. These concentrations, while significant, remain lower than those reported herein.

Furthermore, Gedikoğlu^[^ ^17^ ^]^ found the highest total phenolic content in ethanolic extracts at 132.99 mg GAE g^−1^, whereas flavonoids reached 10.93 mg QE g^−1^, highlighting the substantial impact of solvent type on extract composition.

Palaiogiannis^[^ ^18^ ^]^ reported that a 50% ethanol: water leaf extract contained 95.33 mg GAE g^−1^ of phenolics, while the highest flavonoid content was observed in acetone extracts 28.03 mg QE g^−1^. These results further underscore the role of solvent selection and plant part in determining bioactive compound yield.

The observed seasonal variations have significant implications for pharmaceutical and nutraceutical applications. The spring‐summer period appears optimal for harvesting *C. creticus* for applications requiring high phenolic content (spring) or elevated flavonoid concentrations (summer). This seasonal optimization could enhance the standardization of plant‐based therapeutics and improve the reproducibility of bioactive compound yields.

Based on these comparisons, our results demonstrate markedly elevated values compared with previous reports, supporting the hypothesis that the timing of harvest particularly during spring and summer alongside the selected plant part and local environmental conditions, plays a central role in the accumulation of phenolic and flavonoid compounds. Nonetheless, while the association with environmental factors such as UV exposure and temperature is plausible, the precise mechanistic basis remains to be fully elucidated. Future research combining phytochemical and environmental monitoring will help establish causality more robustly.

These findings also align with the assertions of reference,^[^ ^10^ ^]^ which emphasize the influence of environmental and climatic factors on the dynamic chemical profiles of medicinal plants. This highlights the importance of adopting precise seasonal and technical monitoring strategies to enhance the exploitation of plant resources for therapeutic and pharmaceutical applications.

Furthermore, the substantial phenolic and flavonoid contents observed across all seasons suggest that *C. creticus* represents a valuable source of bioactive compounds year‐round, with seasonal harvesting strategies potentially tailored to specific therapeutic applications.

### 2.2 Antioxidant Activity of Cistus creticus Extracts across the Four Seasons

Plant‐derived phytochemicals are characterized by complex structures and considerable diversity, making it difficult to assess the antioxidant activity of plant extracts using a single assay. This is due to the varying chemical interactions and oxidative inhibition mechanisms among the different constituents.^[^ ^19^ ^]^

**Table** **2** presents the antioxidant activities of ethanolic extracts of *Cistus creticus* collected across the four seasons, assessed using DPPH^**•**^, ABTS^+•^, FRAP, ADS, and SNP assays. Two main indicators were adopted to evaluate efficacy: IC_50_ and A_0.5_ values. Lower values indicate a stronger ability to neutralize free radicals. This antioxidant activity is primarily attributed to the abundance of polyphenolic compounds, known for their hydrogen‐ and electron‐donating properties, singlet oxygen quenching capabilities, and metal chelation potential. Previous studies such as that of Rice‐Evans et al.^[^ ^20^ ^]^ have demonstrated a strong correlation between polyphenolic content and antioxidant efficacy, highlighting their relevance as bioactive indicators in therapeutic application

**Table 2:** Antioxidant activity of *Cistus creticus* extracts across the four seasons.

	IC_50_ [µg mL^−1^]a)						A_0.5_ [µg mL^−1^]			
	DPPH^•^	SE	ABTS^+•^	SE	ADS	SE	FRAP	SE	SNP	SE
C Spring	14.90^a^ ± 0.97	0.56	1.80^c^ ± 0.35	0.20	24.84^a^ ± 2.39	1.37	1.66^c^ ± 0.03	0.017	7.05^a^ ± 0.16	0.09
C Winter	12.66^b^ ± 0.29	0.16	2.88^a^ ± 0.53	0.30	26.66^c^ ± 0.72	0.41	1.68^a^ ± 0.60	0.34	9.93^c^ ± 0.11	0.063
C Autumn	16.28^a^ ± 0.71	0.40	3.22^b^ ± 0.81	0.46	26.22^d^ ± 2.59	1.49	1.95^d^ ± 0.26	0.15	5.43^b^ ± 0.51	0.29
C Summer	6.72^c^ ± 0.07	0.40	2.20^a^ ± 0.30	0.17	2.11^b^ ± 0.19	0.10	1.65^b^ ± 0.06	0.034	11.00^d^ ± 0.29	0.16
BHA*	6.89^a^ ± 0.12	0.069	1.91^d^ ± 0.09	0.05	NT	NT	NT	NT	NT	NT
Acid ascorbic*	22.11^a^ ± 0.78	0.45	14.15^b^ ± 1.1	0.63	NT	NT	6.77^a^ ± 1.15	0.66	7.14^b^ ± 0.05	0.028
Tannic acid*	NT	NT	NT	NT	3.125^c^ ± 0.003	0.001	NT	NT	NT	NT

a) Results are expressed as mean ± standard deviation (SD). Different letters (a, b, c) within a column indicate statistically significant differences (*p* < 0.05). IC_50_: concentration required to inhibit 50% of radical activity; A_0.5_: concentration corresponding to an absorbance of 0.5. NT: not tested. *: means References Standards.

The ethanolic leaf extracts of *Cistus creticus* demonstrated strong antioxidant activity in all the evaluated assays, including DPPH•, ABTS^+•^, FRAP, ADS, and SNP, across the four seasons.

### 2.3 ABTS^+•^ Radical Scavenging Activity

The ABTS^+•^ assay revealed remarkable antioxidant performance, with IC_50_ values ranging from 1.80 ± 0.35–3.22 ± 0.81 μg mL^−1^ across the seasonal *Cistus villosus* extracts. The spring extract exhibited the highest activity, recording the lowest IC_50_ value, thus outperforming BHA (1.91 ± 0.09 μg mL^−1^) and closely approaching the efficacy of BHT (1.25 ± 0.35 μg mL^−1^). In contrast, the autumn extract showed relatively lower activity; however, all seasonal extracts remained within a range considered highly active, highlighting their competitive ability to scavenge ABTS^+•^ radicals.

The low IC_50_ values obtained in this study underscore the remarkable antioxidant efficacy of the seasonal *Cistus creticus* extracts, which clearly surpass previously reported activities in the literature. For example, Atsalakis et al.^[^ ^21^ ^]^ reported an IC_50_ value of 56.2 ± 0.8 μg mL^−1^ for a *Cistus creticus* extract cultivated in Greece, a figure that is significantly higher than that of even our least active extract (summer), highlighting a marked difference in free radical scavenging potential. Similarly, Abu‐Orabi^[^ ^4^ ^]^ documented an IC_50_ of 20.0 μg mL^−1^ for a methanolic extract of *C. creticus*, which, although more active than several other reported values, remains notably less potent than any of our seasonal extracts.

In a recent multidirectional study on three *Cistus* species with ethnomedicinal relevance, Yagi et al.^[^ ^22^ ^]^ described the antioxidant activity of methanolic extracts of *Cistus creticus* leaves as good. However, a direct comparison with our ABTS^+•^ ‐based results (632.73 ± 10.81 mg TE g^−1^) is hindered by the use of different measurement units and assay protocols, which preclude accurate quantitative comparison of antioxidant potency.

Additionally, Mastino et al.^[^ ^23^ ^]^ reported an IC_50_ of 27.012 μg mL^−1^ for an ethyl acetate fraction of *C. creticus* from Sardinia, again indicating significantly weaker activity than any of the extracts evaluated in the present study. Even when compared to related species, such as the *Cistus villosus* extract studied in reference,^[^ ^24^ ^]^ which exhibited an IC_50_ of 1.41 μg mL^−1^—our autumn extract demonstrated nearly tenfold higher antioxidant capacity, further emphasizing the outstanding efficacy of the seasonal extracts investigated herein.

### 2.4 DPPH^•^ Radical Scavenging Activity

In the DPPH^**•**^ assay, the summer extract showed the highest activity (IC_50_ = 6.72 ± 0.07 μg mL^−1^), followed by the winter and autumn extracts, while the spring extract exhibited the weakest activity (14.90 ± 0.97 μg mL^−1^). Comparatively, the reference antioxidants BHT (6.16 ± 0.42) and BHA (6.89 ± 0.12) outperformed all extracts except the summer one, which showed nearly equivalent activity. For context, Carev et al.^[^ ^25^ ^]^ reported an IC_50_ of 0.52  mg mL^−1^ (520 μg mL^−1^) for an aqueous extract—substantially weaker than our results. Likewise, Lahcen et al.^[^ ^26^ ^]^ reported a value of 2.53 mg mL^−1^ for an aqueous leaf extract, indicating lower efficacy than our ethanolic extracts.

In contrast, Waed et al.^[^ ^16^ ^]^ in southern Syria reported, high activity for a methanolic extract (0.009 ± 0.0006 mg mL^−1^ or 9 μg mL^−1^), which outperformed all our extracts except that of summer. Meanwhile, Palaiogiannis et al.^[^ ^18^ ^]^ reported an IC_50_ of 350.99  μg mL^−1^ for an ethanolic extract—substantially less effective than all seasonal extracts in our study. In another study,^[^ ^27^ ^]^ the antioxidant activity of three medicinal plants—*Urtica dioica* (nettle), *Sideritis euboea*, and *Cistus creticus* (rock rose)—was evaluated using their aqueous extracts. The results showed that the *Cistus creticus* extract exhibited the highest antioxidant activity among the tested samples, with the lowest IC_50_ value in the DPPH• assay recorded at 0.20 ± 0.01 mg mL^−1^ (i.e., 200 μg mL^−1^), indicating a good capacity to scavenge free radicals. In comparison, our seasonal ethanolic extracts demonstrated significantly greater efficacy, with the lowest IC_50_ observed in the summer extract at only 6.72 ± 0.07 μg mL^−1^, reflecting a markedly stronger antioxidant activity. This considerable difference is attributed to the type of solvent used, as well as the influence of the harvesting season and geographical location, highlighting the superiority of seasonal ethanolic extracts in enhancing the plant's antioxidant potential.

### 2.5 Superoxide Radical Scavenging Activity (Alkaline DMSO Method)

In the ADS assay, all extracts exhibited comparable efficacy, with IC_50_ values ranging from 24.84 ± 2.39 to 26.66 ± 0.72 μg mL^−1^, which is relatively less effective compared to tannic acid (IC_50_ < 3.125 μg mL^−1^), indicating limited activity in this assay. Bayraktar et al.^[^ ^28^ ^]^ reported the highest antioxidant activity (487 μg AAE mg^−1^ dry matter) in Izmir using an ethanolic leaf extract collected between Oct. and Dec., attributed to active phenolic compounds such as catechin, rutin, and gallic acid.

### 2.6 Reducing Power (RP) Assay

In the FRAP assay, A_0.5_ values for *Cistus creticus* extracts ranged between 1.65 ± 0.06 and 1.95 ± 0.26 μg mL^−1^, with the summer extract showing the highest reducing capacity (lowest A_0_._5_), followed by spring and winter, while the autumn extract displayed the lowest. Compared to the reference compound ascorbic acid (6.77 ± 1.15 μg mL^−1^), all seasonal extracts exhibited much higher reducing capacity, underscoring the strong electron‐donating ability of *Cistus creticus* phenolics and supporting their use as potent natural antioxidants. For comparison, Carev et al.^[^ ^25^ ^]^ reported a value of 0.78 μg mL^−1^ while Palaiogiannis et al.^[^ ^18^ ^]^ reported 1103.11 μmol AAE g^−1^. Despite differing units, our results clearly indicate strong reductive potential.

### 2.7 Silver Nanoparticle‐Based Antioxidant Assay

In the SNP assay, IC_50_ values ranged from 5.43 ± 0.51 to 11.00 ± 0.29 μg mL^−1^. The autumn extract showed the highest activity (lowest IC_50_, outperforming ascorbic acid (7.14 ± 0.05 μg mL^−1^), which highlights its high capacity to scavenge nitric oxide radicals. Conversely, the summer extract exhibited the lowest activity. This study highlights the promising antioxidant potential of *Cistus creticus* extracts, particularly in the ABTS^+•^ assay, where very low IC_50y_ values were recorded, outperforming those in many previous studies. The DPPH• assay also confirmed strong activity, especially for the summer extract, supporting the plant's role as a valuable natural source of antioxidants. Seasonal variations clearly influenced the biological activity; however, no single season consistently dominated across all assays. The summer extract excelled in DPPH• and FRAP, while the spring extract led in ABTS^+•^, and the autumn extract was most effective in inhibiting nitric oxide radicals (SNP). ADS results were relatively similar across seasons.

These findings suggest that the antioxidant efficacy of *Cistus creticus* depends more on the type of assay used than on the season of collection. This variation likely reflects changes in the concentration and composition of bioactive compounds under environmental and phenological influences. Therefore, selecting the optimal harvest season should be tailored to the specific bioactivity targeted, in order to maximize the plant's therapeutic and nutritional value.

### 2.8 Estimation of Inhibitory Activity on α‐Amylase Enzyme

The *α*‐amylase enzyme is a primary target in natural therapeutic strategies for type 2 diabetes due to its role in the enzymatic breakdown of complex carbohydrates into absorbable simple sugars. Inhibiting this enzyme contributes to slowing glucose absorption in the intestines, thereby helping to regulate postprandial blood sugar levels. Numerous studies have demonstrated that plant extracts rich in phenolic compounds and flavonoids possess promising *α*‐amylase inhibitory activity.^[^ ^29^ ^]^ In this context, the seasonal extracts of *Cistus creticus* were evaluated to determine their ability to inhibit this enzyme, serving as an indicator of their potential effectiveness as a natural antihyperglycemic agent (**Table** **3**).

**Table 3:** IC_50_ (µg mL^−1^) values for alpha‐amylase inhibitory activity of *Cistus creticus* extracts from different seasons.

Specificationa)	Alpha‐amylase		
	(IC_50_ µg mL^−1^)		SE
Ethanolic extract	C Spring	368.94^c^ ± 5.54	3.19
	C Winter	493.65^b^ ± 4.19	2.41
	C Autumn	341.92^c^ ± 0.87	0.50
	C Summer	302.61^d^ ± 4.32	2.49
Standard	Acarbose	3650.93^a^ ± 10.70	6.17
LSDp_0.05_	–	–	–
Mean	–	–	–

a) Values represent mean ± standard deviation (SD) of three independent experiments. Different superscript letters (a–c) within a column indicate statistically significant differences between means (*p* < 0.05, one‐way ANOVA followed by post hoc test). SE: standard error; IC_50_: concentration required to inhibit 50% of *α*‐amylase activity.

Based on the values presented in the table, the inhibitory efficacy of *Cistus creticus* extracts collected during the four seasons was evaluated against the *α*‐amylase enzyme and compared with two reference compounds: acarbose (for *α*‐amylase) and thiourea (for urease). The results showed that the IC_50_ values for *α*‐amylase inhibition ranged between (302.61 ± 4.32) and (493.65 ± 4.19) µg mL^−1^, while the IC_50_ value of the reference compound Acarbose was (3650.93 ± 10.70) µg mL^−1^. This indicates that all extracts exhibited significantly higher inhibitory activity than acarbose, particularly the summer extract (C Summer), which demonstrated the strongest inhibitory effect, exceeding that of acarbose by more than tenfold. This highlights its remarkable potential as a natural *α*‐amylase inhibitor. Conversely, the winter extract (C Winter) showed the lowest relative activity among the seasons, although it still remained more effective than the control.

Due to the absence of prior studies specifically investigating the *α*‐amylase inhibitory potential of *Cistus creticus*, our results were compared with another species of the same genus, *Cistus salviifolius*, as reported in the study by Sayah.^[^ ^30^ ^]^ In that study, the IC_50_values of the plant extracts were around 0.42 mg mL^−1^, which renders the activity of *Cistus creticus*, particularly the summer extract (302.61 µg mL^−1^), clearly superior to that of *Cistus salviifolius*, indicating a stronger inhibitory capacity toward *α*‐amylase.

Furthermore, a study by El Hachlafi et al.,^[^ ^31^ ^]^ on *Cistus ladanifer* using essential oil extracted via microwave‐assisted hydrodistillation revealed various biological activities, but did not report a notably high specific activity against *α*‐amylase compared to the strong inhibitory effect observed with *Cistus creticus* extracts in our study. This further supports the functional value of *Cistus creticus* extract as a promising natural source of enzyme inhibitors relevant to blood sugar regulation.

### 2.9 Inhibitory Activity against Urease Enzyme

Urease is an enzyme secreted by numerous microorganisms, and it contributes to the enzymatic hydrolysis of urea into ammonia and carbon dioxide, leading to an increase in environmental alkalinity. The activity of this enzyme has been associated with various health disorders, particularly gastric ulcers caused by *Helicobacter pylori* and urinary tract infections linked to stone formation. Therefore, inhibiting this enzyme is considered an effective therapeutic strategy to limit microbial proliferation and prevent associated complications.^[^ ^32^ ^]^ In this context, *Cistus creticus* extracts were tested to assess their ability to inhibit urease activity. **Table** **4** shows the IC_50_ (50% inhibitory concentration) values for urease‐inhibiting activity of *Cistus creticus* extracts collected in different seasons and the standard inhibitor thiourea. A lower IC_50_ value indicates a stronger inhibitory activity.

**Table 4:** IC_50_ (µg mL^−1^) values for urease inhibitory of *Cistus creticus* extracts from different seasons.

Specificationa)		Antiurease	
		IC_50_ µg mL^−1^	SE
Ethanolic extract	C Spring	66.61^c^ ± 0.75	0.43
	C Winter	96.82^a^ ± 1.25	0.72
	C Autumn	75.66^b^ ± 3.89	2.24
	C Summer	14.41^d^ ± 0.10	0.057
Standard	Thiourea	11.57^d^ ± 0.68	0.39
LSDp_0.05_	–	3.68	–
Mean	–	40.29	–

a) Values are expressed as mean ± standard deviation (SD, *n* = 3). Different superscript letters (a–d) within a column indicate statistically significant differences among samples at *p* < 0.05 (one‐way ANOVA followed by post hoc test). SE: Standard error.

The summer extract (C Summer) showed the strongest urease‐inhibiting activity (IC_50_ = 14.41 µg mL^−1^). This value is marked with the letter “d” and is statistically significantly lower (i.e., the activity is higher) than in all other seasons. The activity of the summer extract is very similar to that of the standard inhibitor thiourea (IC_50_ = 11.57 µg mL^−1^). The fact that both are marked with the letter “d” suggests that there is no statistically significant difference between them, which highlights the important efficacy of the summer extract (Table 4).

Spring extract (C Spring) ranks second in terms of efficacy (IC_50_ = 66.61 µg mL^−1^), marked with the letter “c”, indicating a statistically significantly higher IC_50_ (weaker activity) than summer extract and thiourea, but stronger than autumn and winter extracts. Autumn extract (C Autumn) shows intermediate activity (IC_50_ = 75.66 µg mL^−1^), marked with the letter “b”, significantly different from spring and winter Table 4).

Winter extract (C Winter) has the weakest urease‐inhibiting activity (IC_50_ = 96.82 µg mL^−1^), marked with the letter “a”, making it statistically the least effective of all seasonal extracts Table 4). There is a clear seasonal relationship: Urease inhibition efficiency is highest in summer and lowest in winter, with intermediate values in spring and autumn.

These findings clearly demonstrate a seasonal influence on the biological activity of the plant extracts, with peak activity recorded during the summer season against both *α*‐amylase and urease enzymes. This supports the hypothesis that environmental conditions affect the accumulation of bioactive compounds in the plant.

In the absence of prior studies specifically addressing the urease inhibitory potential of *Cistus creticus*, our results were compared with those reported by Biglar et al.^[^ ^33^ ^]^ who conducted a screening of the effects of extracts from 20 medicinal plants used in Iranian traditional medicine on urease activity. Among these plants, the inhibitory activity of the *Cistus creticus* summer extract surpassed that of at least fifteen species in terms of lower IC_50_ values, including *Thymus vulgaris* (IC_50_ = 68.57 µg mL^−1^), *Cichorium intybus* (IC_50_ = 91.21 µg mL^−1^), and *Matricaria chamomilla* (IC_50_ = 89.75 µg mL^−1^). Although some plants such as *Zingiber officinale* (IC_50_ = 48.54 µg mL^−1^) and *Nigella sativa* (IC_50_ = 59.10 µg mL^−1^) demonstrated notable activity, the summer extract of *Cistus creticus* outperformed all of them, except for the two plants closest to the activity of thiourea.

This comparison highlights the high competitive efficacy of *Cistus creticus* extract as a natural urease inhibitor, potentially exceeding the inhibitory activity of most commonly studied medicinal plants. These findings support its promising potential as a source for the development of anti‐urease agents for future therapeutic applications.

### 2.10 Seasonal Extracts of Cistus creticus and Their Sun Protection Potential

The use of sunscreen products represents an effective and increasingly popular strategy to protect against the harmful effects of ultraviolet (UV) radiation from sunlight. The effectiveness of these products largely depends on the ability of bioactive compounds to absorb, reflect, or scatter UV rays. Formulations exhibiting high sun protection factor (SPF) values are considered more effective in preventing sunburn, as they offer a higher level of photoprotection, as previously reported.^[^ ^34^ ^]^

**Table** **5** shows the SPF values for *Cistus creticus* extracts collected in different seasons, compared with two commercial sunscreens (Nivea, Vichy). A higher SPF value indicates better protection against UV radiation. The letters (a, b, c) indicate statistically significant differences between groups.

**Table 5:** SPF values of *Cistus creticus* extracts from different seasons.

Specification	SPF	SE
C Spring	32.24^a^ ± 0.67	0.38
C Winter	27.76^b^ ± 0.39	0.22
C Autumn	24.95^bc^ ± 1.06	0.61
C Summer	33.94^a^ ± 0.26	0.15
Niveaa)	50.10 ± 0.53	0.30
Vichya)	44.22 ± 0.34	0.19
LSDp_0.05_	5.45	–
Mean	29.72	–

a) Values are expressed as mean ± standard deviation (SD, *n* = 3). Different superscript letters (a–c) indicate statistically significant differences among seasonal means at *p* < 0.05 (one‐way ANOVA followed by LSD post hoc test). *Nivea and Vichy were used as reference sunscreens*. SE: Standard error.

The summer extract (C Summer) has the highest SPF (33.94). This value is marked with the letter “a” and is statistically equivalent to the spring extract, but significantly higher than the winter and autumn extracts. The spring extract (C Spring) also has a high SPF (32.24). Marked with the letter “a”, indicating that it is not statistically different from the summer extract.

The winter extract (C Winter) has an SPF of 27.76, marked with the letter “b”. It is statistically different from the summer and spring extracts (it has a lower SPF), but is not significantly different from the autumn extract. The autumn extract (C Autumn) has the lowest SPF (24.95), marked with the letters “bc”. This value is not significantly different from the winter extract (b), but is statistically lower than the spring (a) and summer (a) extracts. The seasonal dependence is clear: summer and spring extracts offer the highest SPF protection, while autumn extract provides the lowest (Table 5).

*Cistus creticus* extracts have a significant photoprotective potential, which is strongly dependent on the harvest season. Summer and spring extracts are the most effective in protecting against UV radiation, classifying themselves in the “high protection” category according to the European Commission guidelines (SPF ≥ 30). Winter and autumn extracts offer “moderate protection”. These results emphasize that for the optimal use of *Cistus creticus* as a natural ingredient in sunscreen formulations, it is preferable to harvest the plant in summer or spring.

When compared to the reference standards recommended by the European Commission in 2009,^[^ ^34^ ^]^ the extracts fall within the “moderate” to “high” protection categories. According to this classification, formulations with SPF values between 15 and 50 are considered to provide moderate to high protection. Based on this criterion, the summer extract qualifies as offering “high protection” (SPF ≥ 30), as does the spring extract. In contrast, the autumn extract is categorized under “moderate protection” (SPF 15–29.9).

When benchmarked against commercial sunscreens, *Cistus creticus* extracts provided lower SPF than Nivea (50.10) and Vichy (44.22), but their values remain noteworthy for a crude natural extract, particularly since they approach the high protection threshold.

The use of mineral blockers such as zinc oxide or synthetic chemical filters such as ethylhexyl methoxycinnamate is what allows Nivea and Vichy to attain respective SPF ratings, as demonstrated by a direct comparison. The ethanolic extracts of the leaves demonstrated remarkable antioxidant and anti‐inflammatory activities, in addition to satisfying these requirements for the effectiveness of UV blocking.

The plant produced more phytochemical substances to defend itself from UV radiation due to regular exposure to sunshine in the region's climate, which improved its photoprotective ability.

These results highlight the seasonal influence on the accumulation of photoprotective bioactive compounds in *Cistus creticus*, underscoring the importance of harvest timing in enhancing the plant's UV‐protection capacity. This study emphasizes the relevance of selecting the optimal season for plant collection, as the extracts harvested during summer and spring demonstrate greater efficacy in shielding against UV radiation compared to those collected in autumn.

### 2.11 Antimicrobial Activity of Ethanolic Extracts of Cistus creticus across the Four Seasons

This study aimed to evaluate the antimicrobial activity of ethanolic extracts of *Cistus creticus* harvested during the four seasons (autumn, winter, spring, and summer), using the disc diffusion method against four bacterial strains—two Gram‐positive and two Gram‐negative—as well as the fungal strain *Candida albicans* (**Table** **6**).

**Table 6:** Inhibition zone diameters (mm) of ethanolic extracts of *Cistus creticus* harvested in different seasons against selected microbial strains.

Microbial Straina)	Season	10 mg mL^−1^	20 mg mL^−1^	40 mg mL^−1^	80 mg mL^−1^	GNT (10 µg disc^−^^1^)
*Staphylococcus aureus ATCC 25932*	Autumn	10	10	12	15	32
	Winter	8	8	12	14	31
	Spring	14	15	17	18	32
	Summer	11	13	15	17	32
*Bacillus subtilis ATCC 25973*	Autumn	8	8	9	12	24
	Winter	NI	7	8	9	24
	Spring	9	9	13	14	24
	Summer	8	9	10	11	24
*Escherichia coli ATCC 25922*	Autumn	NI	NI	NI	NI	27
	Winter	NI	NI	NI	NI	27
	Spring	NI	NI	NI	NI	27
	Summer	NI	NI	NI	NI	27
*Pseudomonas aeruginosa ATCC 27853*	Autumn	NI	NI	NI	NI	26
	Winter	NI	NI	NI	NI	26
	Spring	NI	NI	NI	NI	26
	Summer	NI	NI	7	8	26
*Candida albicans ATCC 10231*	Autumn	NI	NI	NI	NI	–
	Winter	NI	NI	NI	NI	–
	Spring	NI	NI	NI	NI	–
	Summer	NI	NI	NI	NI	–

a) Notes: NI = No inhibition observed; GNT = Gentamicin (10 µg disc^−^^1^) used as antibacterial control; (–) = Not applicable for fungal strain. Values marked with the same letter are not statistically different from each other.

#### 2.11.1 Activity against Gram‐Positive Bacteria

All extracts demonstrated clear inhibitory effects against *Staphylococcus aureus* and *Bacillus subtilis*, with inhibition zones varying by season and concentration. The spring extract (EXTRAIT Ci/Sp) exhibited the highest antibacterial activity, with an inhibition diameter of 18 mm against *S. aureus* at the highest concentration tested (80 mg mL^−1^), compared to 15 mm in autumn, 14 mm in winter, and 17 mm in summer. Similarly, it showed the most pronounced effect against *B. subtilis* (14 mm), suggesting a higher abundance of bioactive compounds in spring‐harvested material.

In general, inhibition zones decreased gradually with decreasing concentrations, highlighting a clear dose–response relationship (Table 5).

#### 2.11.2 Activity against Gram‐Negative Bacteria

None of the extracts exhibited activity against *Escherichia coli* at any concentration or season, indicating a notable resistance of this strain. However, *Pseudomonas aeruginosa* was weakly inhibited only by the summer extract, with inhibition zones of 8 mm at 80 mg mL^−1^ and 7 mm at 40 mg mL^−1^. No activity was recorded for this bacterium in other seasonal extracts. This suggests a possible presence of seasonal compounds with weak and selective activity against Gram‐negative bacteria.

#### 2.11.3 Antifungal Activity (Candida albicans)

No antifungal activity was observed against *Candida albicans* across all extracts and concentrations, which may reflect either the absence of antifungal compounds or their presence at sub‐effective levels.

#### 2.11.4 Comparison with the Reference Antibiotic (Gentamicin)

Compared to the reference antibiotic gentamicin, the plant extracts displayed considerably weaker activity. Gentamicin produced inhibition zones ranging from 24 to 32 mm, underscoring its potent antibacterial properties and validating its role as a reliable positive control in antimicrobial susceptibility testing.

The absence of antifungal activity against *Candida albicans* in our study is consistent with the findings of Mastino et al.^[^ ^23^ ^]^ who reported that acidic methanolic extracts of *Cistus creticus* from Sardinia exhibited no activity against *Candida albicans*. Moreover, these authors observed a higher efficacy against Gram‐positive bacteria compared to Gram‐negative ones, which supports our observation of weak activity against *Pseudomonas aeruginosa* and *E. coli*.

Similarly, a systematic review conducted by Zalegh^[^ ^8^ ^]^ indicated that essential oils from *Cistus creticus* were effective against *Staphylococcus aureus* and *Bacillus subtilis*, while *E. coli* and *P. aeruginosa* displayed higher resistance. These findings reinforce our results showing the marked resistance of Gram‐negative bacteria. In contrast, a recent study by Agca et al.^[^ ^35^ ^]^ demonstrated that *Cistus creticus* extracts showed significant antibacterial activity against *Bacillus subtilis*, *Bacillus licheniformis*, and *Bacillus amyloliquefaciens*, in addition to exhibiting biofilm inhibitory effects against *Pseudomonas aeruginosa* and *E. coli*. These findings indicate greater efficacy compared to our results, particularly regarding the inhibition of *P. aeruginosa*, which in our case was limited and season‐dependent. When compared to the reference antibiotic gentamicin, our extracts showed considerably weaker activity. This is in agreement with Gedikoğlu et al.^[^ ^17^ ^]^ who reported that aqueous and methanolic extracts of *Cistus creticus* from Turkey exhibited low activity against tested bacterial strains, including Gram‐negative species, thereby explaining the substantial efficacy gap compared to standard antibiotics. Conversely, Atsalakis and Chinou^[^ ^21^ ^]^ reported broad‐spectrum antimicrobial activity of pollen extracts from *Cistus creticus* collected in Greece, with low MIC values ranging from 1.98 × 10^−3^ to 2.98 × 10^−3^ mg mL^−1^. These results clearly surpass our findings and may be attributed to differences in plant material, environmental conditions, and extraction protocols. In a related study, Tomás‐Menor et al.^[^ ^36^ ^]^ showed that *Cistus salviifolius* extracts were effective against *S. aureus*, and that removal of polar fractions enhanced activity against *E. coli*, suggesting that chromatographic fractionation may improve extract efficacy. Together, these comparisons suggest that seasonal variation, extraction strategy, and phytochemical composition critically influence antimicrobial efficacy. Future studies incorporating MIC determinations and fractionation approaches will be essential to optimize the antimicrobial potential of *Cistus* extracts.

### 2.12 Anti‐Inflammatory Activity Determination

Medicinal plants are a rich source of biologically active compounds that have shown promising anti‐inflammatory effects, making them a natural and safe alternative to conventional chemical drugs, which may be associated with undesirable side effects. The importance of using plants in this context lies in their content of compounds such as phenolics, flavonoids, terpenoids, and alkaloids, which act through multiple mechanisms, including the inhibition of inflammation‐inducing enzymes (such as COX and LOX) and the reduction of pro‐inflammatory cytokine production. Moreover, the abundance of these plants, their accessibility, and their ability to adapt to various environmental conditions further support their potential in the development of effective natural therapeutic agents.^[^ ^37^ ^]^

#### 2.12.1 In Vitro Anti‐Inflammatory Activity

In vitro experiments represent a fundamental initial step in evaluating the anti‐inflammatory efficacy of compounds before proceeding to in vivo studies. According to the guidelines of the Organisation for Economic Co‐operation and Development (OECD)^[^ ^38^ ^]^ these in vitro models are used to determine the biological activity of plant extracts or chemical compounds through specific mechanisms, such as the inhibition of inflammatory enzymes (e.g., COX‐2 and 5‐LOX), or the reduction of inflammatory mediators (such as interleukins and prostaglandins) in cultured immune cells. This step aims to verify both the effectiveness and potential toxicity of the compound under controlled conditions, thereby contributing to reducing the need for animal testing in the early stages of research and paving the way for more advanced studies using in vivo models.

The model of bovine serum albumin (BSA) denaturation inhibition was used as a basis to evaluate the potential anti‐inflammatory effects of the four different *Cistus creticus* extracts. The effect of each plant extract from the four seasons on the thermal denaturation of BSA was measured at varying concentrations. The results are organized and presented in **Table** **7** below.

**Table 7:** IC_50_ (µg mL^−1^) values for in vitro anti‐inflammatoryactivity of *Cistus creticus* extracts from different seasons.

		IC_50_ [µg mL^−1^]	SE
Ethanolic extract	C Spring	953.22^b^ ± 0.90	0.51
	C Winter	512.97^a^ ± 3.17	1.83
	C Autumn	313.15^a^ ± 2.74	1.58
	C Summer	385.10^c^ ± 3.9	2.25
Standard	Diclofenac	40.90^d^ ± 0.89	0.51

Results are expressed as mean ± standard deviation (SD). Different letters (a, b, c, d) within a column indicate statistically significant differences (*p* < 0.05).

The anti‐inflammatory activity of *Cistus creticus* extracts from different seasons shows a notable variation in the ability to inhibit the thermal denaturation of BSA. According to the data in the table, the extracts are ranked based on their potency as follows: the winter extract (C Winter) exhibits the lowest IC_50_ value (512.97 µg mL^−1^), indicating the strongest anti‐inflammatory effect compared to the other seasons. In contrast, the autumn extract (C Autumn) shows the highest IC_50_ value (3313.15 µg mL^−1^), demonstrating significantly lower activity in inhibiting BSA thermal denaturation.

The summer extract (C Summer) shows a moderate IC_50_ value (385.10 µg mL^−1^), indicating reasonable anti‐inflammatory activity, while the spring extract (C Spring) ranks second with a higher IC_50_ value (953.22 µg mL^−1^), suggesting less efficacy compared to the other seasons. When comparing the results with Diclofenac, a reference compound in these experiments, it is evident that the *Cistus creticus* extracts exhibited lower effects than Diclofenac, with the IC_50_ values for the plant extracts being higher than that of Diclofenac (40.90 µg mL^−1^).

This variation among the seasons highlights the importance of determining the optimal timing for extracting bioactive compounds from plants, as anti‐inflammatory properties may be more potent in certain seasons compared to others. It emphasizes the influence of environmental factors and climatic conditions on the efficacy of plant extracts and underscores the necessity of utilizing extracts from specific seasons to achieve optimal therapeutic effects.

#### 2.12.2 In Vivo Anti‐Inflammatory Activity

The anti‐inflammatory activity of medicinal plant extracts has been extensively documented in vivo, particularly using the carrageenan‐induced paw edema model in animals, which is considered a standardized and reliable method for assessing acute inflammation. This model is utilized to evaluate the ability of bioactive compounds derived from plants—such as flavonoids, phenolic acids, and terpenoids to inhibit the production or action of inflammatory mediators like prostaglandins and cytokines.^[^ ^39^ ^]^ Although in vitro bioactivity assays represent an important preliminary step for identifying the pharmacological potential of plant extracts, in vivo evaluation remains more accurate and representative of the complex physiological conditions within living organisms. In vivo studies account for factors such as absorption, distribution, metabolism, and excretion, making them essential for confirming the actual efficacy and safety of plant extracts.^[^ ^40^ ^]^

Following carrageenan injection, the control group exhibited a significant paw edema of 51.80 ± 1.92 (**Table** **8**). In contrast, administration of Diclofenac at a dose of 500 mg kg^−1^ reduced the edema to 14.00 ± 1.58. Similarly, treatment with the spring extract of *Cistus creticus* at the same concentration resulted in an edema thickness of 15.80 ± 1.48. When the extract concentration was reduced to one‐fifth, the edema level was still effectively decreased to 19.00 ± 1.58. These results indicate that the anti‐inflammatory efficacy of the plant extract is comparable to that of the synthetic drug, which, despite its effectiveness, is known to cause adverse side effects in living organisms, whereas the extract maintained substantial activity even at a lower concentration.

**Table 8:** Anti‐inflammatory activity results of the spring extract of *Cistus creticus* on carrageenan‐induced paw edema in mice.

Treatment	Dose [mg kg^−1^ b.w]	Edema percentage [%]	Inhibition percentage [%]
Control (saline)	–	51.80^a^ ± 1.92	–
Diclofenac	500	14.00^c^ ± 1.58	72.97
*Cistus creticus* Spring extract	100	19.00^a^ ± 1.58	63.32
	500	15.80^b^ ± 1.48	69.49

Results are expressed as mean ± standard deviation (SD). Different letters (a, b, c) within a column indicate statistically significant differences (*p* < 0.05).

When comparing the anti‐inflammatory activity results of the spring extract of *Cistus creticus* with previous studies on other species of the same genus, a consistent effectiveness is observed. In the current study, the extract at a concentration of 500 mg kg^−1^ reduced paw edema thickness to 15.80 ± 1.48, which is comparable to the reference drug diclofenac (14.00 ± 1.58). Even when the concentration was reduced to one‐fifth, the extract still showed notable efficacy (19.00 ± 1.58).

These findings are in agreement with previous research on other *Cistus* species. For instance, aqueous extracts of *Cistus salviifolius* and *Cistus monspeliensis* demonstrated significant anti‐inflammatory activity in the carrageenan‐induced paw edema model, showing 91.57% and 85.78% inhibition, respectively, 6 h post‐injection.^[^ ^41^ ^]^ Similarly, *Cistus albidus* extract exhibited 76.1% inhibition after 6 h of treatment.^[^ ^42^ ^]^

This study by Eileen Mac et al. emphasizes the anti‐inflammatory potential of *Cistus monspeliensis* L. aerial parts and root extracts with a comparative analysis. Both extracts exhibited a protective effect against the LPS inflammatory stimulus on a macrophage cell line (RAW264.7) at concentrations ranging from 1.56 to 6.25 g mL^−1^.^[^ ^43^ ^]^ These comparisons highlight the potential of different *Cistus* species extracts as natural alternatives to conventional anti‐inflammatory drugs.

### 2.13 Analgesic Activity

Medicinal plants are an important source for the discovery of compounds with analgesic properties. Numerous plant species have shown promising pain‐relieving potential in various studies, mainly due to their content of active phenolic and terpenoid compounds. These compounds act through multiple mechanisms, including modulation of inflammatory and neural pathways involved in pain perception, making them potential natural alternatives to conventional analgesic drugs.^[^ ^44^ ^]^ Based on this, the analgesic activity of *Cistus creticus* extract collected during the spring season was evaluated in this study **Table** **9**.

**Table 9:** Results of the analgesic activity of the spring‐season extract of *Cistus creticus*.

Treatment	Dose [mg kg^−1^]	Number of writhes	Percentage of protection [%]
Control	–	69.00^b^ ± 3.16	–
Paracetamol	500	20.40^c^ ± 1.94	70.43
*Cistus creticus* Sp	100	28^a^ ± 1.58	59.42
	500	19.6^b^ ± 1.81	71.59

Results are expressed as mean ± standard deviation (SD). Different letters (a, b, c) within a column indicate statistically significant differences (*p* < 0.05).

The results of this study demonstrated a notable analgesic activity of *Cistus creticus* extract prepared during the spring season. Pain inhibition reached 59.42% at a dose of 100 mg kg^−1^ and increased to 71.59% at 500 mg kg^−1^. This effect slightly surpassed that of paracetamol, used as a positive control at the same concentration (70.43%), indicating the potential of the extract as a natural analgesic. This activity may be attributed to the presence of phenolic and terpenoid compounds known for their ability to inhibit inflammatory mediators and modulate peripheral neural pathways involved in pain perception.

These findings are consistent with those reported by Ait Lahcen,^[^ ^26^ ^]^ who showed that *Cistus creticus* leaf extract exhibits antioxidant and antimicrobial properties—traits that serve as indirect indicators of possible activity on inflammatory and pain‐related pathways. Due to the lack of direct previous studies on the analgesic effect of this plant, our results were compared with those from studies on other plants. For example, Qnais et al.^[^ ^45^ ^]^ reported that *Artemisia herba‐alba* extract exhibited both analgesic and anti‐inflammatory effects in animal models, supporting the reliability of using plant‐based extracts for pain relief.

Similarly, the findings of Quintans Júnior et al.^[^ ^46^ ^]^ indicated that *Salvia officinalis* extract had a clear analgesic effect in experimental pain models, which aligns with the significant activity observed in our study with *Cistus creticus* . Together, these results reinforce the potential of this plant as a promising source for the development of effective and safe natural alternatives to conventional analgesic drugs.

### 2.14 Acute Toxicity

No mortality was observed among the mice exposed to increasing doses of the spring‐season extract of *Cistus creticus*, up to the maximum tested dose of 2000 mg kg^−1^ body weight. Throughout the 14‐day observation period, animals did not display any behavioral or clinical manifestations of toxicity, including hyperactivity, ataxia, tremors, convulsions, salivation, diarrhea, lethargy, drowsiness, or coma. Body weight gain, food, and water intake remained within the normal range. These findings suggest that the extract is relatively safe at the administered dose and does not exhibit evident acute toxicity. This assessment was conducted in accordance with the OECD Guideline No. 423 (2002) 423,^[^ ^47^ ^]^ for acute oral toxicity testing. The LD_50_ of the extract was, therefore, estimated to be over 2000 mg kg^−1^.

### 2.15 Descriptive Statistics

**Table** **10** shows descriptive statistics for nine different parameters studied in ethanolic extracts of *Cistus creticus* (TPC, TFC, antioxidant activities: DPPH•, ABTS^+•^, ADS, FRAP, SNP; SPF; and enzymatic/therapeutic activities: anti‐inflammatory, antiurease, antialpha‐amylase).

**Table 10:** Descriptive statistics of TPC, TFC, antioxidants, SNP, SPF, anti‐inflammatories, antiurease, antialpha‐amylase.

Specification	TPC	TFC	DPPH^•^	ABTS^+•^	ADS	FRAP	SNP	SPF	Anti‐inflam	Antiurease	Antialfa amylase
Mean	609.30	233.08	12.64	2.53	19.96	1.74	8.35	29.72	541.11	376.78	63.38
Median	602.26	247.63	13.60	2.32	25.21	1.68	8.53	29.41	425.45	353.15	69.85
Standard deviation	35.57	40.40	3.85	0.74	10.89	0.31	2.33	4.11	260.59	74.74	31.71
Kurtosis	−0.78	−0.59	−0.85	−0.83	−0.43	1.11	−1.77	−1.15	−0.73	−0.70	−0.75
Skewness	0.66	−1.01	−0.80	0.51	−1.23	0.77	−0.15	−0.02	1.00	0.90	−0.80
Range	100.28	104.96	10.45	2.33	26.72	1.14	6.24	12.58	653.14	199.99	83.88
Minimum	567.13	167.35	6.64	1.43	1.89	1.22	5.00	22.87	300.95	297.95	14.34
Maximum	667.41	272.31	17.10	3.76	28.61	2.35	11.24	35.45	954.09	497.94	98.22
Va) [%]	5.84	17.33	30.46	29.37	54.59	17.76	27.92	13.84	48.16	19.84	50.03

a) Coefficient of variation.

The following measures were calculatedfor each parameter: 1) Mean: The central value of the data. 2) Median: The middle value in an ordered set of data, less sensitive to outliers than the mean. 3) Standard deviation: A measure of the spread of the data around the mean. A higher deviation indicates greater variation in the results. 4) Kurtosis: A measure of the shape of the distribution, indicating the “sharpness” of the distribution peak and the “thickness” of its tails. Negative values indicate a flatter distribution (fewer extreme values), and positive values indicate a more “spiky” distribution (more extreme values). 5) Skewness: A measure of the asymmetry of the data distribution. Positive values indicate a right‐skewed distribution (tail on the right, mean > median), and negative values indicate a left‐skewed distribution (tail on the left, mean < median). 6) Range: The difference between the maximum and minimum values, indicating the overall range of the observed data. 7) Minimum: The lowest observed value. 8) Maximum: The highest observed value. 9) Coefficient of variation (V %): * A percentage measure of the relative dispersion of the data (standard deviation divided by the mean, multiplied by 100%). A higher coefficient indicates greater relative variability.

This Table 10 allows for a quick assessment of the statistical characteristics of each of the measured parameters, including their typical values, dispersion, distribution shape, and variability. Of particular interest are the coefficients of variation, which indicate which parameters exhibit the greatest relative differences in the sample of extracts studied.

Summary of variability assessment: Lowest variability: TPC (5.84%) shows a very low relative variability. This means that total phenolic content is the most stable parameter in the extracts tested, regardless of the factors that differentiate the samples (e.g., season, although this study focused on seasons, so these results provide a general picture of variability in the entire seasonal sample). ‐Moderate variability (10–20%): TFC (17.33%); FRAP (17.76%); SPF (13.84%); antiurease (19.84%). These parameters show a reasonable level of variability, which is typical for many biological and chemical characteristics in plant extracts. High and very high/extremely high variability (>20%): DPPH• (30.46%), ABTS^+•^ (29.37%), SNP (27.92%), ADS (54.59%) ‐ The highest variability was shown by anti‐inflammatory (48.16%) and antialpha amylase (50.03%). Antioxidant tests (especially ADS, DPPH•, ABTS^+•^, SNP) and enzymatic/therapeutic activities (anti‐inflammatory and antialpha amylase) show very high relative variability. This means that the results for these activities vary greatly between the tested samples. This is consistent with previous results, which indicated a strong effect of season on these activities (e.g., summer extract was a much stronger alpha‐amylase inhibitor). The high variability of these parameters suggests that they are very sensitive to factors such as harvest season, plant growth conditions, or extraction methods. The data indicate that while total phenolic content is relatively stable, biological activities (antioxidant and enzyme‐inhibiting) show much greater relative variability in *Cistus creticus* extracts. This high variability is a key finding that highlights the importance of controlling factors such as season of harvest to obtain extracts with predictable and desirable biological properties.

### 2.16 Analysis of Statistical Significance of Pearson Correlation—description

**Figure 1:**
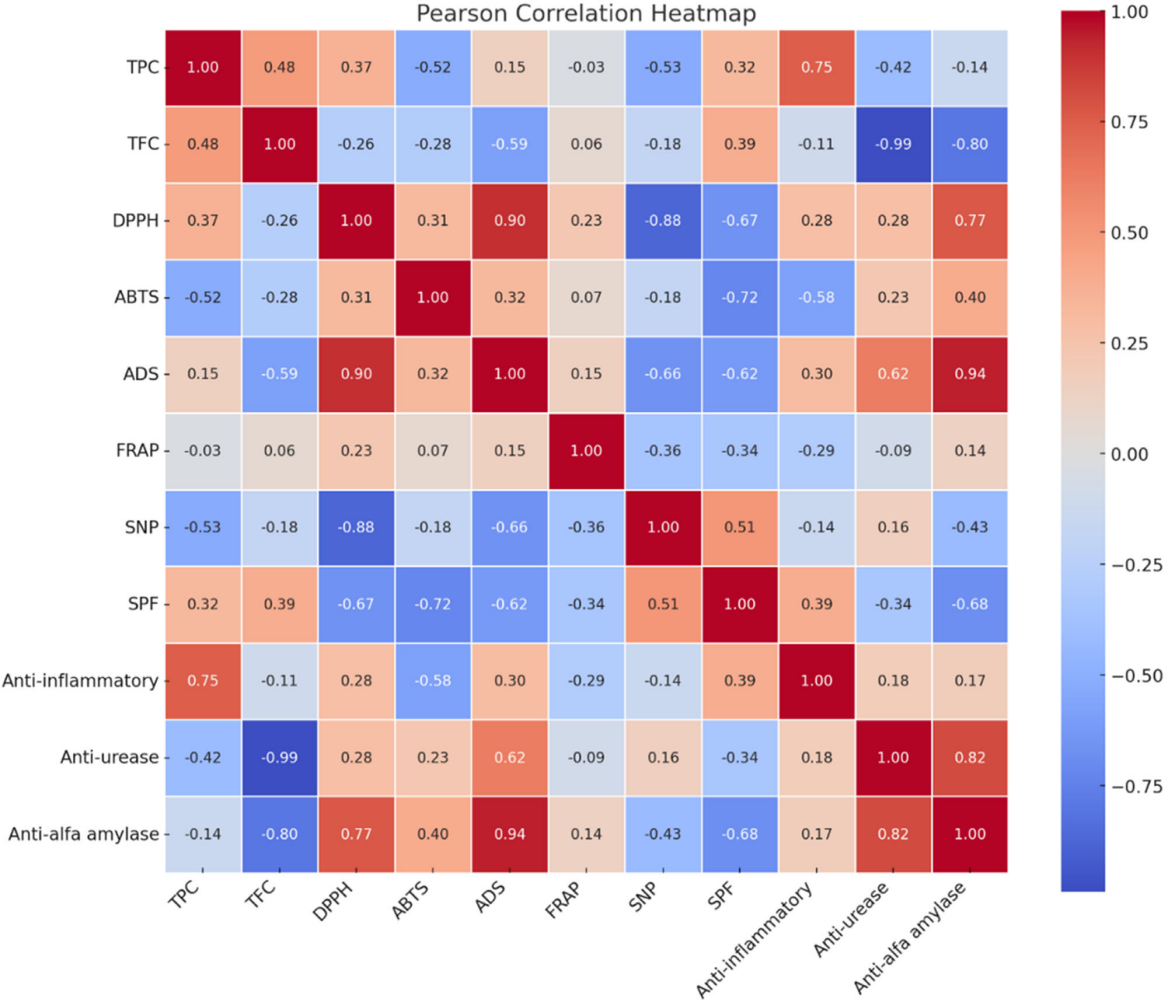
Visualization of the Pearson correlation matrix in the form of a heat map.

Aim of the analysis: The aim was to check which relationships between the parameters studied (such as TPC, TFC, DPPH•, SPF, anti‐inflammatory activity, etc.) are statistically significant, i.e., unlikely to result solely from chance. The relationships in the Pearson correlation matrix are briefly described, based on the strength and direction of the coefficients. Values close to +1 indicate a strong positive correlation (both variables increase/decrease together), values close to −1 indicate a strong negative correlation (one variable increases, the other decreases), and values close to 0 indicate no linear correlation (**Figure** **1**).

Key observations include: 1) Relationships between TPC and TFC and antioxidant activity (DPPH•, ABTS^+•^, ADS, FRAP, SNP): a) TPC : there is a moderate positive correlation with DPPH• (*r* = 0.37) and ADS (*r* = 0.15). b) There is also a moderate negative correlation with ABTS^+•^ (*r* = −0.52) and SNP (*r* = −0.53). There is no correlation with FRAP (*r* = −0.03). Hence, the conclusion: Phenolic content has a complex effect on antioxidant activity depending on the test, which highlights the complexity of the mechanisms of antioxidant action. c) TFC: There is a weak negative correlation with DPPH• (*r* = −0.26), ABTS^+•^ (*r* = −0.28), ADS (*r* = −0.59), and SNP (*r* = −0.18), and a weak positive correlation with FRAP (*r* = 0.06). Hence, the conclusion: Flavonoids showed mostly a weak, negative correlation with antioxidant activity in these tests, which may suggest that other phenolic compounds (nonflavonoids) or synergistic effects of ingredients are more responsible for this activity, or that individual flavonoids have a different effect. ‐ Correlations in antioxidant activity: Strong positive correlation between DPPH• and ADS (*r* = 0.90): Indicates that these assays measure similar aspects of antioxidant activity. d) Strong negative correlation between DPPH• and SNP (*r* = −0.88): Suggests that compounds that are active in the DPPH• assay may be less active in the SNP assay and vice versa, or that these assays assess different mechanisms. e) Strong negative correlation between ADS and SNP (*r* = −0.66): Suggests a similar observation to the above. Therefore, antioxidant activity was multifaceted; different assays may assess different mechanisms and types of compounds. 2) Correlations with antiurease and antialpha‐amylase activity: a) TFC showed a strong negative correlation with antiurease (*r* = −0.99) and antialpha‐amylase (*r* = −0.80), meaning that the higher the flavonoid content, the stronger the inhibitory effect on these enzymes (lower IC_50_ values indicate more substantial effect, so a negative correlation with IC_50_ is desirable). This was a very strong indication that flavonoids are the key compounds responsible for inhibiting urease and alpha‐amylase. ADS (superoxide radical scavenging activity) has a strong positive correlation with antiurease (*r* = 0.62) and antialpha‐amylase (*r* = 0.94): This indicates that the compounds responsible for scavenging the superoxide radical (measured by ADS) are also strong inhibitors of urease and alpha‐amylase. b) DPPH• showed a strong positive correlation with antialpha‐amylase (*r* = 0.77): Strong activity in the DPPH• test goes hand in hand with potent inhibition of alpha‐amylase. 3) Correlations with anti‐inflammatory activity (Anti‐infl) and SPF: a) TPC had a strong positive correlation with Anti‐infl (*r* = 0.75): Higher phenolic content is associated with stronger anti‐inflammatory effects. b) TFC showed a moderate positive correlation with SPF (*r* = 0.39): Flavonoids contributed to sun protection. c) ABTS^+•^ showed a strong negative correlation with SPF (r = −0.72): The stronger the scavenging activity of ABTS^+•^, the lower the SPF, which is counterintuitive and may suggest specific mechanisms or components influencing these properties. d) SNP showed a moderate positive correlation with SPF (0.51): The ability to scavenge nitric oxide radical correlates positively with SPF (Figure 1).

## 3 Conclusion

This study demonstrates that seasonal variation profoundly influences the phytochemical profile and biological activities of *Cistus creticus*, with spring and summer extracts showing the highest pharmacological promise. Their richness in phenolics and flavonoids was reflected in potent antioxidant, enzyme inhibitory, anti‐inflammatory and photoprotective effects.

These findings underline the importance of harvest timing for standardizing bioactive yields and optimizing the therapeutic, nutraceutical, and cosmeceutical potential of *C. creticus*. While the work was limited to a single site without detailed climatic data, the consistent seasonal trends observed provide a compelling basis for future multilocation studies integrating metabolomics and environmental parameters. Further isolation and mechanistic studies of active metabolites will be critical to validate their pharmacological relevance.

Overall, this work establishes *C. creticus* as a versatile natural resource and highlights the need to integrate seasonal and ecological factors into cultivation and product development strategies to ensure reproducibility, efficacy, andsustainable use.

## 4 Experimental Section

4.1

4.1.1

##### Chemicals and Reagents

All solvents used in this present study were of analytical grade obtained from Honeywell, 1,1‐diphenyl‐1‐picrylhydrazyl (DPPH), Folin‐Ciocalteu reagent, aluminum chloride (AlCl_3_ 6H_2_0), sodium carbonate (Na_2_CO_3_), Ferrozine, potassium ferricyanide, ammonium molybdate (NH_4_)_6_Mo_7_O_24_ 4H_2_0, 2,2′‐azinobis (3‐ethylbenzothiazoline‐6‐sulfonic acid) diammonium salt (ABTS^+•^), sodium molybdate (Na_2_MoO_4_), sodium acetate (C_2_H_3_NaO_2_), (Sigma–Aldrich GmbH, Stern‐heim, Germany. The reference standards were obtained from EXTRASYNTHESE (Genay Cedex, France). These standards included Quercetin (C_15_H_10_O_7_), butylated hydroxytoluene (BHT) (C_15_H_24_O), gallic acid (C_7_H_6_O_5_) butylated hydroxyanisole (BHA) (C_11_H_16_O_2_), ascorbic acid (C_6_H_8_O_6_), EDTA, and alpha‐tocopherol.

##### Sample Collection and Preparation of Extracts

Leaves of *Cistus creticus* were collected during the mid‐point of each season (autumn, winter, spring, and summer) between 2022 and 2023 from the El Kef Lakhdar area in Medea Province, Algeria (35°57′33″N, 3°12′16″E; 1,164 m altitude). The species was authenticated by the Department of Chemistry, University of El Oued, and a voucher specimen (Cis‐cre‐003‐1‐2022) was deposited in the Herbarium of the Laboratory of Biomass, ENS‐Kouba, Algiers, Algeria.

Only healthy plants growing under similar environmental conditions were selected for this study. To minimize variability due to phenological stage, only fully expanded, mature leaves were collected, while young or senescent leaves were excluded. Fresh material was transported in clean airtight containers, washed thoroughly with deionized water, and air‐dried in the shade at 20–25 °C for 15 days in a well‐ventilated environment to prevent photodegradation. The dried material was then ground to a fine powder (<200 µm) using an electric grinder and stored in airtight containers at room temperature until extraction.

For each seasonal extract, 15 g of powdered leaf material was macerated with 500 mL of ethanol–water (8:2, v/v) at room temperature for 24 h under continuous mechanical stirring. The mixture was filtered, and the extraction was repeated three times to ensure exhaustive recovery. The combined filtrates were concentrated under reduced pressure at 40 °C using a rotary evaporator. The crude extracts were stored in amber glass bottles at 4 °C until further analysis.

The percentage of yield of crude extract was calculated using the following equation:

(1) Yield %=(Weight of dried crude extracts (g)/Weight of dried plant sample taken (g))×100

##### Termination of Total Phenolic and Flavonoid Contents: TPC

The TPC in the ethanolic extracts of *Cistus creticus* was quantified using a microplate‐based protocol derived from the Folin–Ciocalteu method, following the procedure outlined by Müller et al.^[^ ^48^ ^]^ Specifically, 20 µL of each extract was combined with 100 µL of a tenfold‐diluted Folin–Ciocalteu reagent and 75 µL of a 7.5% sodium carbonate solution. The mixture was incubated in the dark at room temperature for two hours. Absorbance was measured at 765 nm using a microplate spectrophotometer. Gallic acid served as the reference compound to construct the standard calibration curve (*y* = 0.0034*x* + 0.1044, *R* ^2^ = 0.9972). Results were reported as micrograms of gallic acid equivalents per milligram of extract (µg GAE mg^−1^ extract).

##### Termination of Total Phenolic and Flavonoid Contents: TFC

Flavonoid concentration was determined through a colorimetric method adapted from Topçu et al.^[^ ^49^ ^]^ A volume of 50 µL of ethanolic extract was mixed with 130 µL of methanol, 10 µL of 1 M potassium acetate, and 10 µL of 10% aluminum nitrate. Following a 40‐min incubation period in the dark at ambient temperature, absorbance was recorded at 415 nm. Quercetin was used as the standard reference (*y* = 0.0048*x*, *R* ^2^ = 0.997), and flavonoid content was expressed as micrograms of quercetin equivalents per milligram of extract (µg QE mg^−1^ extract).

##### Evaluation of Biological Activities: DPPH• Radical Scavenging Activity

The antioxidant potential of *Cistus creticus* ethanolic extracts was assessed through their ability to neutralize the DPPH• (2,2‐diphenyl‐1‐picrylhydrazyl) free radical, following the procedure described by Silva.^[^ ^50^ ^]^ A 0.1 mM DPPH• solution was prepared in methanol, and 160 µL of this solution was added to 40 µL of either the plant extract or standard antioxidants (BHA or BHT). After vigorous agitation, samples were incubated at room temperature in the dark for 30 min. Absorbance was then read at 517 nm. Radical scavenging activity was quantified as IC_50_, the concentration required to inhibit 50% of DPPH• radicals.

##### Evaluation of Biological Activities: ABTS^+•^ Radical Scavenging Activity

The capacity of the extracts to scavenge ABTS^**+•**^ (2,2′‐azino‐bis(3‐ethylbenzothiazoline‐6‐sulfonic acid)) radicals was determined according to the method of Zhao et al.^[^ ^51^ ^]^ ABTS radicals were generated by mixing 2 mM ABTS^+•^ with 2.45 mM potassium persulfate and allowing the mixture to stand in the dark for 16 h. The solution was then diluted to an absorbance of 0.700 ± 0.025 at 734 nm. A total of 40 µL of extract was added to 160 µL of the ABTS^+•^ solution, and absorbance was measured at 734 nm. Results were expressed as IC_50_ values.

##### Evaluation of Biological Activities: RP Assay

The ferric‐reducing antioxidant power of the ethanolic extracts was evaluated by measuring their ability to convert Fe^3+^ to Fe^2+^in accordance with the procedure developed by Jiménez‐Morales et al.^[^ ^52^ ^]^ Briefly, 10 µL of each extract was combined with 40 µL of 0.2 M sodium phosphate buffer (pH 6.6) and 50 µL of 1% potassium ferricyanide. After incubation at 50 °C for 20 min, the absorbance was recorded at 700 nm. RP was expressed as the extract concentration required to reach an absorbance of 0.5 (A_0.5_).

##### Evaluation of Biological Activities: Superoxide Radical Scavenging Activity (Alkaline DMSO Method)

The superoxide scavenging effect of the extracts was investigated using the alkaline DMSO technique as proposed by Tsaplev et al.^[^ ^53^ ^]^ The assay mixture consisted of 30 µL of 1 mg mL^−1^ NBT, 40 µL of the test extract, and 130 µL of alkaline DMSO (containing DMSO, 5 mM NaOH, and water). Absorbance was determined at 560 nm, and the IC_50_ values were calculated to express the superoxide scavenging activity.

##### Evaluation of Biological Activities: Silver Nanoparticle‐Based Antioxidant Assay

An additional antioxidant evaluation was conducted using the silver nanoparticle (AgNP) formation method described by Savitri et al.^[^ ^54^ ^]^ A colloidal AgNP solution was prepared by heating 50 mL of 1 mM AgNO_3_ for 10 min, followed by the gradual addition of 5 mL of 1% sodium tri‐citrate until a pale‐yellow color appeared. For the assay, 130 µL of AgNP solution and 50 µL of distilled water were mixed with 20 µL of the extract. The mixture was incubated at 25 °C for 30 min, and absorbance was measured at 423 nm. Antioxidant activity was reported as the concentration required to attain an absorbance of 0.5 (A_0.5_).

##### α‐Amylase Inhibition Assay

The inhibitory effect of *C. creticus* ethanolic extracts on *α*‐amylase activity was examined using the iodine–potassium iodide colorimetric method, following the modified protocol of Bouchareb et al.^[^ ^55^ ^]^ A mixture of 25 µL of extract and *α*‐amylase enzyme (prepared in sodium phosphate buffer, pH 6.9, containing 6 mM NaCl) was incubated at 37 °C for 10 min. Then, 50 µL of a 1% starch solution was added, followed by further incubation for 20 min. The reaction was stopped by adding 25 µL of 1 M HCl, and color development was achieved by adding 100 µL of iodine–potassium iodide reagent. Absorbance was measured at 630 nm, and the percentage of enzyme inhibition was calculated using appropriate controls.

##### Urease Inhibition Assay

The potential of the extracts to inhibit urease enzyme activity was assessed using the phenol–hypochlorite method. Each reaction included 10 µL of extract, 25 µL of urease enzyme solution (5 U mL^−1^), and 50 µL of 17 mM urea. Following a 50‐min incubation at 30 °C, 45 µL of phenol reagent and 70 µL of alkaline reagent were added. Absorbance was recorded at 630 nm, and IC_50_ values were calculated. Thiourea was used as the standard inhibitor.

##### SPF Determination

The photoprotective capacity of the extracts was evaluated in vitro using the spectrophotometric method described by Kaur et al.^[^ ^56^ ^]^ Extracts were diluted in methanol to a final concentration of 2 mg mL^−1^. Absorbance was recorded across the UVB spectrum (290–320 nm) at 5 nm intervals using a multimode microplate reader. SPF values were calculated using the formula:

(2) $$\mathrm{SPF}=\mathrm{CF}\times \sum_{290}^{320}\mathrm{EE}\left(\lambda \right)\times I\left(\lambda \right)\times \mathrm{Abs}\left(\lambda \right)$$

where CF is the correction factor (10), EE(*λ*) is the erythemal effect spectrum, I(*λ*) is the solar intensity spectrum, and Abs(*λ*) is the sample absorbance at each wavelength.

##### Antibacterial Activity

The antimicrobial activity of the ethanolic extracts was investigated using the disc diffusion method. Sterile filter paper discs (6 mm in diameter) were loaded with 35 µg of the extract and placed on Mueller–Hinton agar plates inoculated with test microorganisms: *Staphylococcus aureus* (ATCC 6538), *Bacillus subtilis* (ATCC 6633), *Pseudomonas aeruginosa* (ATCC 9027), *Escherichia coli* (ATCC 8739), and *Candida albicans* (ATCC 10,231). Standard antibiotics (fosfomycin, carbenicillin, erythromycin, and cephalexin) served as positive controls. Plates were incubated at 37 °C for 48 h, and zones of inhibition were measured in millimeters.

##### In Vitro Anti‐Inflammatory Activity

The anti‐inflammatory potential of the extracts was assessed by their capacity to prevent thermal denaturation of BSA, following the method of Benmohamed et al.^[^ ^57^ ^]^ Each reaction mixture contained 1 mL of extract or diclofenac (reference drug) and 1 mL of 0.2% BSA in Tris‐HCl buffer (pH 6.6). The mixtures were incubated at 37 °C for 15 min and then heated to 72 °C for 5 min. After cooling, absorbance was measured at 660 nm. Inhibition percentage was calculated with reference to untreated controls.

##### In Vivo Anti‐Inflammatory Activity

The anti‐inflammatory activity in vivo was examined using the carrageenan‐induced paw edema model in Swiss albino mice (20–25 g), following the protocol of Whiteley and Dalrymple.^[^ ^58^ ^]^ Animals were divided into three groups (*n* = 5): a control group receiving 0.5 mL of physiological saline, a standard group treated with 10 mg kg^−1^ diclofenac sodium, and a treatment group administered 0.5 mL of 10% *C. creticus* extract. After 30 min, each mouse received a subplantar injection of 0.025 mL of 1% carrageenan into the left hind paw. After 4 h, mice were sacrificed, and paw weights were measured. Edema inhibition was calculated as a percentage relative to the control.

##### Evaluation of Analgesic Activity

Peripheral pain suppression in a test compound can be measured using the famous acetic acid‐induced writhing test in mice. The purpose is to see if the test substance can lessen pain. All mice were grouped into either those that received the test drug or grouped as controls that only receive saline. Following the dose, acetic acid is applied to the animals in their abdomen to cause writhes. fixed duration from 20 to 30 min is allotted for recording writhes when observing pets; protection is determined for each analgesic by applying the following formula, in addition, substances with a higher protection percentage are considered to have better pain‐relieving powers.

(3) Protection %=((Mean writhes in control group−Mean writhes in treated group)/Mean writhes in control group)×100

##### Acute Oral Toxicity Test

Acute toxicity was evaluated according to OECD guideline 423.^[^ ^47^ ^]^ Three groups of fasting mice (*n* = 5 per group) received a single oral dose of *C. creticus* ethanolic extract at 275, 1562, and 2555 mg kg^−1^, respectively. The animals were monitored daily for 14 days for signs of mortality, behavioral abnormalities, or any toxicological symptoms.

##### Statistical Analysis

Data are expressed as mean ± standard deviation (SD) of three independent replicates. Seasonal differences were assessed using one‐way ANOVA, followed by Tukey's HSD post‐hoc test for pairwise comparisons; different letters (a, b, c) indicate significant differences at *p* < 0.05. Normality and variance homogeneity were evaluated using descriptive statistics (skewness, kurtosis, and coefficient of variation) prior to applying ANOVA. Pearson's correlation coefficients were calculated to assess the associations between phytochemical contents and biological activities. When variables deviated from normality, Spearman's rank correlation was used instead. Correlation matrices were visualized as heatmaps. All analyses were performed using STATISTICA 10.0 (StatSoft Inc., USA).

## Conflict of Interest

The authors declare no conflict of interest.

## Author Contributions

**Khalil Guelifet**: writing—review & editing, writing—original draft, visualization, supervision, resources, project administration, methodology, investigation, data curation. **Mokhtar Benmohamed**: visualization, supervision, methodology, investigation. **Khaled Kherraz**: writing—review & editing, writing—original draft, investigation**. Latifa Khattabi**: writing—review & editing, supervision, methodology, investigation. **Barbara Sawicka**: writing—review & editing, supervision, methodology, investigation. **Ayomide Victor Atoki**: writing—review & editing, writing—original draft, visualization, supervision, methodology, investigation, data curation. **Abderrazek Ferhat**: visualization, supervision, investigation. **Lilya Harchaoui**: investigation, methodology. **Mohamed Amine Ferhat**: writing—review & editing, writing—original draft, visualization, supervision, resources, project administration, methodology, investigation, data curation. **Wafa Zahnit**: visualization, supervision, investigation. **Afaf Khadra Bendrihem**: writing—review & editing, writing—original draft, visualization, supervision, methodology, investigation, data curation. **Mohammed Messaoudi**: writing—review & editing, writing—original draft, visualization, supervision, resources, project administration, methodology, investigation, data curation.

## Statement of Human and Animal Rights

This study was conducted in accordance with institutional and international guidelines for the care and use of laboratory animals. All procedures were approved by the appropriate ethics committee and complied with the ethical standards concerning animal welfare. No human participants were involved in this study.

## Data Availability

The data that support the findings of this study are available from the corresponding author upon reasonable request.
